# Prevalence and Associated Factors of Hypercalcemia of Malignancy Among Advanced Cancer Patients Attending Palliative Care Unit of a Tertiary Care Hospital in Bangladesh

**DOI:** 10.1002/cnr2.70157

**Published:** 2025-03-11

**Authors:** Rafsana Rouf, A. K. M. Motiur Rahman Bhuiyan, Afroja Alam, Mostofa Kamal Chowdhury

**Affiliations:** ^1^ Department of Palliative Medicine Bangabandhu Sheikh Mujib Medical University (BSMMU) Dhaka Bangladesh

**Keywords:** hypercalcemia, neoplasm, palliative care, prevalence

## Abstract

**Background:**

Hypercalcemia is a common complication of advanced malignancy and a palliative care emergency. Its incidence increases in the later stages of the disease and is linked to a poor prognosis. Understanding the prevalence, clinical characteristics, and factors associated with malignancy‐related hypercalcemia is crucial for developing targeted interventions and enhancing overall palliative care for advanced cancer patients.

**Aim:**

This study aims to provide baseline data on the prevalence, clinical presentation, and factors associated with hypercalcemia among advanced cancer patients receiving palliative care in Bangladesh.

**Methods:**

This cross‐sectional study was conducted among 155 advanced cancer patients admitted to the Department of Palliative Medicine, Bangabandhu Sheikh Mujib Medical University, Bangladesh, from June to December 2023. Data were collected through patient histories, physical examinations, and laboratory analyses. Statistical tests, including Chi‐square test, Fisher's exact test, and Mann Whitney *U* test (for skewed data), were used to examine associations between socio‐demographic and disease‐related variables and serum calcium levels. Binary logistic regression was applied to identify factors affecting hypercalcemia.

**Result:**

Among the participants, 20% had hypercalcemia, with 51.6% experiencing mild, 25.8% severe, and 22.6% moderate hypercalcemia. Head‐and‐neck cancers (12.9%) and gastrointestinal malignancies (22.6%) were more common in hypercalcemic patients. Lethargy, polyuria, polydipsia, constipation and dehydration were more prevalent among hypercalcemic participants. However, lethargy, nausea, confusion, drowsiness, abdominal pain, and constipation, which are typically more common in hypercalcemic patients, were more frequent in the normocalcemic group. Binary logistic regression analysis revealed that age between 41 and 65 years, male sex, poor performance status, and a short disease duration (up to 6 months) were significant risk factors for developing hypercalcemia among cancer patients.

**Conclusion:**

Malignant hypercalcemia affected nearly one‐fourth of patients with advanced malignancy in our study setting. It is important for healthcare providers to be aware of the common symptoms of hypercalcemia and to regularly monitor calcium levels in advanced cancer patients.

## 1 Background

Hypercalcemia is defined as elevated serum calcium levels exceeding 2.6 mmol/L (10.5 mg/dL) [1]. It affects approximately 1%–2% of the general population and can result from conditions such as primary hyperparathyroidism, thyrotoxicosis, excessive vitamin D intake, certain medications (e.g., thiazide diuretics), and malignancy. Among these, cancer‐associated hypercalcemia is the most common cause [2, 3]. A nationwide analysis in the United Kingdom (UK) estimated that 3%–30% of cancer patients develop hypercalcemia during their illness [4].

The incidence of hypercalcemia in early‐stage cancer is low (1%–5%) [5], but it becomes more common in advanced stages, where it is often linked to a poor prognosis. Patients with hypercalcemia in advanced cancer typically have a median survival of only 40–68 days [6, 7]. Moderate to severe cases present with various symptoms, while mild hypercalcemia may be asymptomatic or cause vague symptoms like fatigue or anorexia, making diagnosis challenging. If left untreated, hypercalcemia can rapidly progress to severe levels, leading to a rapid decline in the patient's condition [8].

Palliative care aims to enhance the quality of life for individuals and their families facing terminal illnesses by addressing physical, psychological, social, and spiritual challenges. Cancer patients frequently experience electrolyte imbalances, including hyponatremia, hyperuricemia, and hypercalcemia, which can contribute to organ dysfunction, increased morbidity, and reduced quality of life. Hypercalcemia often presents with vague symptoms and may go undetected. If untreated, it can lead to severe complications such as fatal cardiac arrhythmias, renal impairment, and even death [4, 9, 10]. Studies indicate that hypercalcemia at any stage of cancer is associated with lower quality of life, while early detection and treatment can significantly improve symptom management and overall well‐being [11].

Currently, there is a lack of studies on the prevalence of hypercalcemia and its associated factors among advanced cancer patients in palliative care settings in Bangladesh. Understanding the prevalence, clinical characteristics, and factors associated with hypercalcemia of malignancy is crucial for developing targeted interventions for cancer patients in the country. This study serves as a baseline investigation into the prevalence, clinical presentation, and factors associated with hypercalcemia among advanced cancer patients attending a tertiary palliative care center in Bangladesh. Although this research focuses on the local context, its findings can contribute to the global understanding of hypercalcemia in advanced cancer patients.

## 2 Methods

### 2.1 Study Design and Setting

This cross‐sectional study was conducted among adult patients (aged ≥ 18 years) diagnosed with stages III and IV cancer, who were admitted to the Department of Palliative Medicine at Bangabandhu Sheikh Mujib Medical University (BSMMU), Shahbag, Dhaka. Data collection took place from June to December 2023.

### 2.2 Sample Size

We used a census method to determine the sample size for this study. All stage III and IV cancer patients admitted to the palliative care ward of BSMMU during the study period were included. From June to December, a total of 330 adult advanced cancer patients were admitted to our department. We approached all of these patients, but 38 refused to provide informed consent, 35 passed away before blood samples could be collected, and 102 had repeated admissions. As a result, the final sample size was 155.

### 2.3 Data Collection Procedure

Data were collected by the principal investigator using a well‐structured questionnaire. The purpose of the study was explained to the patients and their caregivers and/or relatives, and written informed consent was obtained before the interview. Within 24 h of admission, symptoms were assessed based on the presenting complaints obtained from the patients or their caregivers, and signs were elicited through physical examinations and physician‐guided clinical assessments. Additionally, 5 mL of venous blood was collected aseptically in a vacutainer tube by the trained nurses working in the Department of Palliative Medicine. The labeled samples were then sent to the Department of Biochemistry and the Department of Hematology for the required tests. Biochemical analysis included serum calcium, serum albumin, serum electrolytes, and serum creatinine, while the hematological analysis included a complete blood count with ESR. The blood sample for biochemical analysis was analyzed using an Automated Analyzer: Atellica, Siemens, Germany, in the Biochemistry Department, and the hematological analysis was conducted using the SYSMEX 6‐Part Diff. Automated Hematology Analyzer (Model: XN2000) in the Hematology Department. The reports were then collected and noted in the data collection form. The corrected calcium levels of the patients were calculated using the following formula developed by Payne et al. [12]: Corrected Calcium = (0.8 × (Normal Albumin—Pt's Albumin)) + Serum Ca.

Patients with corrected serum calcium level ≥ 10.5 mg/dL were considered hypercalcemic. Corrected serum calcium level between 10.5 and 11.9 mg/dL was classified as mild hypercalcemia, between 12 and 13.9 mg/dL as moderate hypercalcemia, and serum calcium level ≥ 14 mg/dL was considered severe hypercalcemia [3].

### 2.4 Data Analysis

All statistical analyses were performed using SPSS version 26. Continuous variables, such as age, duration of the disease, and biochemical and hematological data, were presented as mean, SD, median, and range. Qualitative variables, such as gender, cancer type, staging, treatment status, hypercalcemia categories, signs, and symptoms, were expressed as frequency, percentage, and graphs.

Chi‐square test and Fisher's exact test were used to assess the association between socio‐demographic, disease, and treatment‐related variables, clinical findings, and different types of hypercalcemia. The association of serum creatinine and GCS score with different types of hypercalcemia was determined using the Mann–Whitney *U* test (for skewed data). Binary logistic regression was performed to identify the factors affecting hypercalcemia among the study population. All means were calculated with a 95% confidence interval, and *p*‐values < 0.05 were considered statistically significant.

### 2.5 Ethical Considerations

The ethical approval (Approval no: BSMMU/2023/8891, Date: June 15, 2023) was obtained from the Institutional Review Board (IRB) of Bangabandhu Sheikh Mujib Medical University, Bangladesh. Written informed consent was taken from all the eligible patients and/or their caregivers.

## 3 Results

Among the 155 participants, 52.9% were male and 47.1% were female, with a male‐to‐female ratio of 1.12:1. The study participants were predominantly aged 41–65 years (58.1%), with an average age of 52.65 ± 13.88 years. Half (51%) of the patients were admitted to the palliative medicine department within 6 months of diagnosis. Most patients were at Stage IV (64.5%) and had a bed‐bound status with ECOG‐4 (55.5%) performance status. Metastasis to lymph nodes was the most common (31%), followed by bone (28.4%), liver (27.7%), and lung (23.2%) metastasis (Table 1).

**TABLE 1:** Distribution of study population according to sociodemographic and disease related characteristics (*n* = 155).

Variables	Frequency	Percentage (%)
Age (in years)		
18–40	38	24.5
41–65	90	58.1
Above 65	27	17.4
Mean ± SD (years)	52.65 ± 13.88	
Sex		
Male	82	52.9
Female	73	47.1
Primary Diagnosis		
Unknown primary	16	10.3
Gastrointestinal system	52	33.5
Respiratory	13	8.4
Head and neck	28	18.1
Hematological	5	3.2
Genitourinary	13	8.4
Breast	23	14.8
Duration since diagnosis (months)		
Upto 6 months	79	51
7–14 months	39	25.1
Above 15 months	37	23.9
Eastern Co‐operative Oncology Group (ECOG) performance status		
Confined to bed more than 50% of waking hours (Grade 3)	69	44.5
Completely bedbound (Grade 4)	86	55.5
Sites of metastasis^a^		
Brain	11	7.1
Lung	36	23.2
Liver	43	27.7
Bone	44	28.4
Lymph node	48	31
Peritoneum	34	21.9
Disseminated	1	0.6

^**a**^ Multiple response.

Among all participants, 31 (20%) had hypercalcemia. Of these hypercalcemic patients, 16 (51.6%) had mild hypercalcemia, 8 (25.8%) had severe hypercalcemia, and 7 (22.6%) had moderate hypercalcemia (Table 2).

**TABLE 2:** Distribution of the study population according to serum calcium level (*n* = 155).

Serum calcium level	Frequency	Percentage (%)
Normocalcemia	124	80
Hypercalcemia	31	20
Severity of hypercalcemia (*n* = 31)		
Mild hypercalcemia	16	51.6
Moderate hypercalcemia	7	22.6
Severe hypercalcemia	8	25.8

Cancers of the head and neck (12.9%) and gastrointestinal systems (22.6%) were more prevalent among hypercalcemic patients. However, head and neck malignancies were significantly more common in these patients compared to the normocalcemic group (7.3%). Additionally, lung metastasis (67.3%) and bone metastasis (58.1%) were significantly more common in hypercalcemic patients than in the normocalcemic group (lung 1.6% and bone 10.5%). Most hypercalcemic patients (70.9%) had a shorter disease duration (up to 6 months) compared to normocalcemic patients (45.9%). Furthermore, a significantly higher proportion of hypercalcemic patients (77.4%) were bedbound compared to normocalcemic patients (50%) (Table 3).

**TABLE 3:** Association of serum calcium status with sociodemographic and disease related variables (*n* = 155).

Variables	Hypercalcemia (*n* = 31)	Normocalcemia (*n* = 124)	*p*
	Frequency (%)	Frequency (%)	
Age (in years)			
18–40	4 (12.9)	34 (27.4)	
41–65	21 (67.73)	69 (55.6)	**0**.**02**
Above 65	6 (19.3)	21 (21.7)	
Gender			
Male	21 (67.7)	61 (49.2)	**0**.**04**
Female	10 (32.3)	63 (50.8)	
Primary diagnosis			
Gastrointestinal system	7 (22.6)	45 (36.2)	
Head and neck	4 (12.9)	9 (7.3)	
Respiratory system	3 (9.7)	25 (20.2)	**0**.**044**
Hematological	0 (0)	5 (4.1)	
Others	17 (54.8)	40 (32.2)	
Sites of metastasis^a^			
Lung	21 (67.3)	2 (1.6)	
Bone	18 (58.1)	13 (10.5)	**0**.**005**
Others	14 (45.2)	80 (64.5)	
Duration since cancer diagnosis (months)			
Upto 6 months	22 (70.9)	57 (45.9)	
7–14 months	3 (9.6)	36 (29.3)	**0**.**029**
Above 15 months	6 (19.3)	31 (25.0)	
Cancer stage			
Stage‐III	8 (25.8)	47 (37.9)	
Stage‐IV	23 (74.2)	77 (62.1)	0.208
Eastern Co‐operative Oncology Group (ECOG) performance status			
Grade 3	7 (22.5)	62 (50.0)	
Grade 4	24 (77.4)	62 (50.0)	**0**.**006**

*Note:* chi square test was done; *p*‐value < 0.05 considered as significant.

^a^ Multiple response.

A significant association was observed between lethargy, confusion, polydipsia, polyuria, nausea, constipation, drowsiness, dehydration, and GCS score with serum calcium status. Lethargy, polyuria, polydipsia, constipation, and dehydration were more prevalent among hypercalcemic participants. However, symptoms such as lethargy, nausea, confusion, drowsiness, abdominal pain, and constipation, which are typically more common in hypercalcemic patients, were more frequent in the normocalcemic group. There was a noteworthy elevation in creatinine levels in the hypercalcemic group (*p* < 0.05) (Table 4).

**TABLE 4:** Association of serum calcium status with clinical presentations and biochemical variables (*n* = 155).

Symptoms*	Hypercalcemia (*n* = 31)	Normocalcemia (*n* = 124)	*p*
	Frequency (%)	Frequency (%)	
Lethargy	31 (100)	62 (50.0)	< **0**.**001** ^a^
Confusion	10 (32.2)	11 (8.9)	**0**.**002** ^b^
Seizure	4 (12.9)	11 (35.5)	0.502^b^
Polydipsia	12 (38.7)	0 (0)	< **0**.**001** ^b^
Polyuria	11 (35.5)	0 (0)	< **0**.**001** ^b^
Pruritus	1 (3.2)	12 (9.7)	0.467^b^
Nausea	19 (61.3)	51 (41.1)	**0**.**044** ^a^
Anorexia	20 (64.5)	66 (53.2)	0.258^a^
Vomiting	13 (41.9)	46 (37.1)	0.620^a^
Abdominal pain	14 (45.2)	69 (55.6)	0.295^a^
Constipation	21 (67.7)	39 (31.5)	< **0**.**001** ^a^
Drowsiness	10 (32.3)	11 (8.9)	**0**.**002** ^b^
Unconsciousness	0 (0)	2 (1.6)	> 0.99^b^
Signs*			
Some dehydration	22 (70.9)	20 (16.1)	< **0**.**001** ^a^
Severe dehydration	8 (25.8)	1 (0.8)	< **0**.**001** ^b^
GCS score (Median (range))			
	10 (3–15)	14 (10–15)	< **0**.**001** ^c^
Biochemical variables	Median	Median	
Creatinine (mg/dL)	1.80	0.80	**0**.**015** ^c^

*Note:* Degree of dehydration was calculated by this formula: Percent dehydration = (pre‐symptom weight − weight during symptom)/pre‐symptom weight × 100. Here, 5%–10% = Some dehydration and > 10% = Severe dehydration.

^a^ Chi square test done.

^b^ Fisher's exact test was done.

^c^ Mann Whitney *U*‐test done.

* *p*‐value < 0.05 considered as significant.

Binary logistic regression analysis revealed that participants aged 41–65 years were twice as likely (OR: 1.667; 95% CI: 0.541–5.140; *p* = 0.373) to develop hypercalcemia compared to other age groups. Similarly, male patients were 1.7 times more likely (OR: 1.701; 95% CI: 0.681–4.250; *p* = 0.256) to experience hypercalcemia than female patients. Those with a disease duration of up to 6 months were also twice as likely (OR: 1.633; 95% CI: 0.543–4.913; *p* = 0.383) to have hypercalcemia. Furthermore, bedbound patients with ECOG grade 4 were more prone to develop hypercalcemia (Table 5).

**TABLE 5:** Factors associated with hypercalcemia by binary logistic regression analysis.

Predictors	OR	95% CI		*p*
		Lower	Upper	
Age (41–65 years vs. other age category)	1.667	0.541	5.140	0.373
Gender (Male vs. Female)	1.701	0.681	4.250	0.256
Disease duration (upto 6 months vs. other duration)	1.633	0.543	4.913	0.383
ECOG (grade 3 vs. other categories)	0.325	0.126	0.838	0.020

*Note:* *p*‐value < 0.05 considered as significant.

Abbreviations: CI, Confidence interval; OR, odds ratio.

## 4 Discussion

Malignant hypercalcemia is considered a palliative care emergency. This study investigates the prevalence, clinical presentations, and contributing factors associated with hypercalcemia of malignancy in the palliative care population.

In our study, 20% of advanced cancer patients were found to have hypercalcemia. This result is consistent with two different studies and a review that report the prevalence of hypercalcemia in advanced cancer patients to range from 3% to 30% [4, 13, 14]. However, the prevalence of hypercalcemia among our patients is much higher than that reported in a large‐scale study conducted in the United States, where the overall prevalence of hypercalcemia was only 2%–3% [15]. The higher prevalence among our population may be attributed to the fact that all of our patients had stage III or IV cancers. A UK‐based study reported that the prevalence of hypercalcemia is four times higher in stage IV cancer [4].

In our study, half of the patients (51.6%) had mild hypercalcemia, while the remaining 48.4% had moderate to severe hypercalcemia. In contrast to our finding, Gupta and colleagues in India reported that only one‐fourth (29.8%) of advanced cancer patients presented with mild hypercalcemia [7].

In our study, participants aged 41–65 were twice as likely to develop hypercalcemia compared to other age groups. This finding aligns with several studies conducted in Thailand over the past 6 years, where hypercalcemia of malignancy (HCM) was commonly observed in patients aged 41 to 60 years. It has been suggested that the increased prevalence of cancer in this age range contributes to the higher number of hypercalcemic cases [16, 17, 18]. However, Wu and colleagues in Taiwan found no significant difference in the prevalence of hypercalcemia across age groups [19].

Similarly, we found that male patients were twice as likely to develop hypercalcemia compared to females. This finding is consistent with research from Taiwan, where females were less prone to hypercalcemia [19]. A study conducted in Thailand by Sookprasert also reported that male patients were more prone to developing hypercalcemia, supporting our results [20].

Our study also found that head and neck, as well as gastrointestinal malignancies, were more prevalent among hypercalcemic patients. Head and neck cancers were significantly more likely to develop hypercalcemia than other malignancies. Our result is consistent with four separate studies from India, Brazil, Taiwan, and Egypt, which found a strong association between head‐and‐neck cancers and hypercalcemia of malignancy (HCM) [6, 7, 19, 21]. A systematic review reported that colorectal cancers were also associated with hypercalcemia [8]. However, several meta‐analyses have found a higher prevalence of hypercalcemia of malignancy (HCM) among patients with hematological malignancies, as well as breast and renal cell carcinomas [8, 22]. We did not observe such associations, possibly due to the limited number of these cancer types in our study population.

We observed that lethargy, polydipsia, polyuria, and dehydration were common in hypercalcemic patients. Our findings align with the review study conducted by Almuradova and Cicin, which identified these symptoms as major clinical presentations in hypercalcemic patients [8]. Several reviews have also reported that neuropsychiatric, gastrointestinal, and renal symptoms are more prevalent among patients with hypercalcemia of malignancy [5, 8, 23].

In our study, hypercalcemic patients had notably elevated creatinine levels compared to the normocalcemic group. Similarly, Gupta et al. found that 36.2% of hypercalcemic patients presented with impaired renal function [7]. Severe hypercalcemia can lead to acute renal impairment, and pre‐existing renal issues may also contribute to the development of hypercalcemia [15, 24]. However, we did not investigate the underlying causes of renal impairment in our study participants.

We also found that symptoms such as lethargy, nausea, confusion, drowsiness, abdominal pain, and constipation, which are commonly seen in hypercalcemic patients, were also more frequent in normocalcemic patients. Although we did not find any specific study addressing such an association, it can be suggested that many of these symptoms may be linked to the patients' primary diseases. Since all our patients had stage III and IV malignancies, and most received multiple anticancer medications as well as morphine, these symptoms can occur even without elevated serum calcium levels. Therefore, all advanced cancer patients need to be carefully assessed, as these symptoms can overlap and sometimes mask the presentation of hypercalcemia.

We also found that a significantly higher proportion of bed‐bound (ECOG‐4) cancer patients presented with hypercalcemia. While the exact mechanism remains largely unclear, the pathogenesis of this condition is primarily associated with a dysregulation between bone resorption and formation. Prolonged bed rest leads to decreased mechanical loading, which tends to increase osteoclast‐induced bone resorption and prompts osteocytes to secrete proteins such as sclerostin, reducing osteoblast‐mediated bone formation. A study conducted in the USA also supported our findings [25]. Additionally, a study by Ramos and colleagues demonstrated a link between poor performance status and hypercalcemia [6].

The duration of primary cancer also contributed to an increased risk of hypercalcemia. In our study, patients diagnosed with cancer within 6 months were twice as likely to develop hypercalcemia. A study on Chinese advanced cancer patients reported that hypercalcemia typically occurs more than 140 days after cancer diagnosis, while Ramos et al. found that it occurred more than 180 days after diagnosis, which aligns closely with our findings [6, 26]. In our study, we found that bone and lung metastases were significantly associated with hypercalcemia among advanced cancer patients. This finding is supported by several studies that have similarly identified these metastases as risk factors for developing hypercalcemia in cancer patients [6, 7, 26].

Three different studies have identified several factors as poor prognostic indicators of hypercalcemia of malignancy (HCM), including age > 40 years, male sex, ECOG performance status greater than 2, metastasis to bone, liver, and kidney, and the presence of neurological symptoms at diagnosis [6, 20, 26]. We also found that the same factors were associated with hypercalcemia. Most of these factors are non‐modifiable. While pharmacological treatments can help lower serum calcium levels in hypercalcemic patients, sustaining a normocalcemic state requires addressing the underlying malignancy [27]. In palliative care, many patients are unable to receive anticancer treatments due to poor performance status. Therefore, early diagnosis of hypercalcemia and prompt treatment with rehydration, bisphosphonates, or denosumab can greatly benefit these patients [14].

### 4.1 Limitations and Recommendations

Our study had several limitations. Firstly, it was conducted in a single tertiary care hospital, which may limit the generalizability of the findings to other palliative care settings across Bangladesh. A multicenter study with a larger sample size would provide a more comprehensive understanding of the prevalence and risk factors associated with hypercalcemia of malignancy in advanced cancer patients. Secondly, while we identified key clinical and demographic risk factors, our study did not explore the molecular or genetic mechanisms underlying hypercalcemia in malignancy. Additionally, we did not assess the patients' lifestyle before admission, which could have contributed to the development of hypercalcemia, such as excessive calcium intake or use of diuretics. Future research on biochemical and genetic profiling, nutrition, medications, and comorbidities could help clarify the pathophysiology of hypercalcemia of malignancy and identify potential biomarkers for early detection. Finally, we did not assess the impact of hypercalcemia on the quality of life and survival outcomes of patients. Longitudinal studies tracking symptom progression, treatment response, and overall prognosis would be valuable in determining the clinical significance of hypercalcemia in advanced cancer patients.

## 5 Conclusion

Hypercalcemia of malignancy is a significant complication among advanced cancer patients, affecting nearly one‐fourth of those in this study. Common symptoms, including lethargy, polydipsia, polyuria, and dehydration, often overlap with those observed in normocalcemic patients. It is important for healthcare providers to be aware of these symptoms and to monitor calcium levels regularly in advanced cancer patients.

## Author Contributions

**Rafsana Rouf:** conceptualization, investigation, data collection, data analysis, writing and editing the manuscript. **A. K. M. Motiur Rahman Bhuiyan:** conceptualiation, funding, supervision, writing and editing manuscript and review. **Afroja Alam:** supervision, writing and review of the manuscript. **Mostofa Kamal Chowdhury:** supervision, writing and review of the manuscript.

## Conflicts of Interest

The authors declare no conflicts of interest.

## Data Availability

All data relevant to the study are available upon request to the corresponding author.
